# Assessment of Newly Proposed Clinical Criteria to Identify *HNF1A* MODY in Patients with an Initial Diagnosis of Type 1 or Type 2 Diabetes Mellitus

**DOI:** 10.1155/2016/4243784

**Published:** 2016-01-28

**Authors:** Malgorzata Grzanka, Bartlomiej Matejko, Magdalena Szopa, Beata Kiec-Wilk, Maciej T. Malecki, Tomasz Klupa

**Affiliations:** Department of Metabolic Diseases, Jagiellonian University Medical College, Jagiellonian University, 15 Kopernika Street, 31-501 Krakow, Poland

## Abstract

The most common form of maturity-onset diabetes of the young (MODY) is caused by mutations in the hepatocyte nuclear factor 1A (*HNF1A*) gene. However, most* HNF1A* mutation-carriers are initially misdiagnosed with type 1 (T1DM) or type 2 (T2DM) diabetes mellitus; hence, they often receive nonoptimal treatment. The aim of our study was to test newly proposed clinical criteria for the identification of* HNF1A* MODY in patients with a diagnosis of T1DM or T2DM. To achieve this, the following criteria to preselect patients for screening were used: for T1DM: TDIR (total daily insulin requirement) > 0.3 IU of insulin/kg and the percentage of basal insulin > 30% of TDIR; for T2DM: sulphonylurea- (SU-) based oral treatment (monotherapy or combined with Metformin) > 15 years and BMI < 30 kg/m^2^. We reviewed the clinical data of 140 patients with T1DM and 524 clinically diagnosed with T2DM. On the basis of these criteria, we found a* HNF1A* mutation in 1 out of 2 individuals with a diagnosis of T1DM and 1 out of 11 selected individuals with a diagnosis of T2DM. We believe that the simplicity of the proposed criteria might prove useful in clinical practice, as an alternative to more time-consuming classical diagnostic techniques.

## 1. Introduction

The most common form of maturity-onset diabetes of the young (MODY) is caused by mutations in the hepatocyte nuclear factor 1A (*HNF1A*) gene [1, 2]. It is well established that patients with* HNF1A* MODY are very sensitive to sulphonylureas (SU) and can be taken off insulin treatment after the proper molecular diagnosis [1, 2]. However, most patients with this type of monogenic diabetes are initially misdiagnosed with either type 1 (T1DM) or type 2 (T2DM) diabetes mellitus; hence, they often receive nonoptimal treatment [3]. The effective selection of “T1DM” and “T2DM” patients for molecular testing for the* HNF1A* MODY mutation does present some challenges. It is usually based on clinical criteria suggested by Ellard et al. [2]. For* HNF1A* MODY they include young-onset diabetes, being non-insulin-dependent outside the normal honeymoon period, family history of diabetes, the absence of pancreatic islet autoantibodies, glycosuria at blood glucose levels <10 mmol/L, marked sensitivity to sulfonylureas, and no marked obesity or evidence of insulin resistance [2].

However, a recent retrospective analysis of a large pediatric cohort showed that only a small portion of MODY subjects were properly diagnosed in spite of having negative pancreatic autoantibodies, preserved endogenous insulin production, or strong family history of diabetes in multiple generations [4]. Biochemical biomarkers are also of limited value [2]. High sensitivity CRP (hsCRP) seemed to be one of the most promising. However recent data suggest that while being a good marker to make differential diagnosis between* HNF1A* MODY and T2DM, it brings no additional value to differentiate between patients with established diagnosis of* HNF1A* MODY and individuals with a clinical history suggestive of* HNF1A* MODY, but not confirmed genetically [5].

*Aim of the Study*. The aim of our study was to test newly proposed clinical criteria for selecting patients with an initial clinical diagnosis of either T1DM or T2DM for* HNF1A* MODY molecular testing.

## 2. Material and Methods

Subjects diagnosed with either T1DM or T2DM were preselected and ascertained as previously defined [6, 7]. All patients underwent molecular testing at the Department of Metabolic Diseases, Medical College, Jagiellonian University, Krakow, Poland [8]. The study was approved by the Bioethical Committee of Jagiellonian University.

### 2.1. Differential Diagnosis: T1DM versus* HNF1A* MODY

To establish the clinical criteria for preselecting individuals diagnosed with T1DM for* HNF1A* molecular testing, we performed a retrospective analysis of patients with an established* HNF1A* MODY diagnosis (131 patients, 46 males and 85 females). We found that before genetic testing 40 patients had been diagnosed and treated as T1DM patients. Based on the characteristics of these 40 misdiagnosed patients, we established new criteria for further molecular testing for* HNF1A* mutations. We subsequently reviewed the clinical data of 140 patients diagnosed with T1DM to preselect individuals meeting our newly established criteria. The diagnosis of T1DM was based on clinical criteria; autoantibodies were not determined.

## 3. Results

### 3.1. T1DM versus* HNF1A*

Analysis of the clinical data of 40* HNF1A* mutation-carriers, who had been treated as T1DM before MODY diagnosis, showed that, on average, their total daily insulin dose was 0.28 j/kg ± 0.11 j (range: 0.19–0.34 j/kg). The data concerning the percentage of basal insulin versus TDIR was available for only six individuals and its median was equal to 32% ± 6 j (range: 26–33%). On the basis of these data, we established the cut-off point for potential MODY at 0.3 IU of insulin per kg and the percentage of basal insulin being >30% of TDIR. As reported earlier, the mean insulin requirement per kg of body mass for T1DM patients was 0.69 IU/kg, whereas the percentage of basal insulin was 42.6% [9].

Of the 140 patients with an initial T1DM diagnosis, based on the aforementioned criteria, two individuals were selected for further molecular testing by Sanger sequencing. A previously described diabetes-related mutation in exon 4 (P291fsinsC) was found in one individual [1]. Interestingly, the maternal family history of this patient was negative for diabetes; however, no data on the paternal branch of the family was available.

### 3.2. T2DM versus* HNF1A*

To select subjects from the T2DM cohort for* HNF1A* gene sequencing, the following criteria were used: SU-based oral treatment (SU in monotherapy or combined with Metformin) for >15 years and a body mass index < 30 kg/m^2^. We analyzed the medical records of 524 patients clinically diagnosed with T2DM to preselect individuals for genetic screening for the* HNF1A* MODY mutation. Based on suggested clinical criteria, we selected 14 subjects from the T2DM patient cohort; however, only 11 were available for testing (9% of selected T2DM cases). In one of these 11 patients, a previously undescribed mutation was identified: a deletion of amino acids 1283–1309 of the* HNF1A* gene. Since this deletion is relatively large (1.3% of exon 7) and is located in the carboxyl-terminal transactivation domain, which is crucial for* HNF1A* function, it is highly probably that this is a causative variant of diabetes in this subject [10]. Retrospective analysis of the clinical data of this patient revealed positive family history for diabetes, which is suggestive of an autosomal-dominant mode of inheritance. Unfortunately, other family members were not available for segregation analysis.

## 4. Discussion

Misdiagnosis of monogenic diabetes has an impact both on glycemic control and on quality of life of affected individuals [1]. There is hope that, with improved accessibility of next generation sequencing (NGS), the number of patients correctly diagnosed with MODY will increase substantially; however, at this point in time, access to this high-throughput methodology is still limited [11]. Here, we propose new clinical criteria for preselection of patients initially diagnosed with either T1DM or T2DM for genetic testing for the identification of mutations in* HNF1A*. As there were some earlier reports suggesting that* de novo* MODY mutations might be more frequent than previously considered, we did not include family history as part of our criteria [12]. Of note, in our study, the* HNF1A* mutation-carrier identified among patients with a clinical diagnosis of T1DM was indeed negative for diabetes in his families. Interestingly, in a recent study involving patients with an initial diagnosis of T1DM, which used three traditional criteria (negative pancreatic autoantibodies, sustained endogenous insulin production, and strong family history of diabetes in multiple generations), only one of 58 patients was identified to be a* HNF1A* mutation-carrier [13].

As for differential diagnosis between T2DM and* HNF1A* MODY, the success rate based on our proposed preselection criteria was relatively small (1 out of 11). This may mean that clinical criteria to identify* HNF1A* mutation-carriers should be probably more restrictive. Testing them would require much larger group of patients to come up with sufficient number of individuals meeting those narrow, more restrictive criteria.

In general, due to relatively small sample size, our study should be considered as a preliminary one. Furthermore, the assessment of diagnostic sensitivity and specificity of our criteria would require sequencing, preferentially NGS, to be performed in the entire initial cohort of T1DM and T2DM subjects.

## 5. Conclusions

We believe that criteria proposed in the present study might be useful for clinicians, as an alternative to classical criteria, to select T1DM and T2DM patients who should undergo molecular testing for mutations in* HNF1A*. Though our criteria show potential, further research and validation are required.
